# Ethyl 4-hy­droxy-1-methyl-5-oxo-2-phenyl­pyrrolidine-3-carboxyl­ate 1.25-hydrate

**DOI:** 10.1107/S1600536813001943

**Published:** 2013-01-26

**Authors:** Nurul Shulehaf Mansor, Mohd Fazli Mohammat, Zurina Shaameri, Hamid Khaledi

**Affiliations:** a Organic Synthesis Labratory, Institute of Science, Universiti Tecknologi MARA, 40450 Shah Alam, Selangor, Malaysia; b Department of Chemistry, University of Malaya, 50603 Kuala Lumpur, Malaysia

## Abstract

The asymmetric unit of the title compound, C_14_H_17_NO_4_·1.25H_2_O, consists of four substituted pyrrolidone mol­ecules (two pairs of enanti­omers) and five water mol­ecules. The five-membered rings each have an envelope conformation, with the C atom bonded to the ester group as the flap. The mean planes of the five-membered rings of the four pyrrolidone mol­ecules make dihedral angles of 60.87 (5), 64.45 (5), 62.03 (5) and 65.79 (5)° with respect to the phenyl rings. In the crystal, the pyrrolidone and water mol­ecules are connected through O—H⋯O hydrogen bonds, forming a layer parallel to the *ab* plane. The two-dimensional network is further stabilized by inter­molecular C—H⋯O hydrogen bonds.

## Related literature

For the structures of similar compounds, see: Ma & Jiang (1998 ▶); Chu *et al.* (2011 ▶); Gainsford & Mason (2010 ▶). For the synthesis of the precursor substituted 2,5-dihydro­pyrrole-2-one, see: Mohammat *et al.* (2009 ▶).
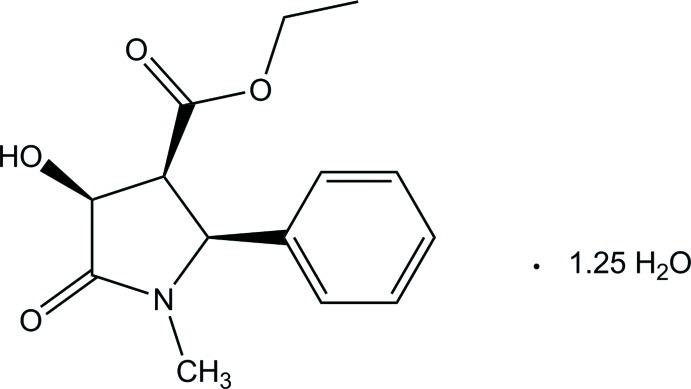


## Experimental

### 

#### Crystal data

C_14_H_17_NO_4_·1.25H_2_O
*M* *_r_* = 285.81
Triclinic, 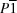

*a* = 10.3669 (2) Å
*b* = 15.7466 (3) Å
*c* = 18.4539 (3) Å
α = 77.373 (1)°
β = 80.425 (1)°
γ = 87.058 (1)°
*V* = 2898.31 (9) Å^3^
*Z* = 8
Mo *K*α radiation
μ = 0.10 mm^−1^
*T* = 100 K
0.21 × 0.15 × 0.09 mm

#### Data collection

Bruker APEXII CCD diffractometer
Absorption correction: multi-scan (*SADABS*; Sheldrick, 1996 ▶) *T*_min_ = 0.979, *T*_max_ = 0.991
22505 measured reflections
11260 independent reflections
9637 reflections with *I* > 2σ(*I*)
*R*_int_ = 0.018

#### Refinement

*R*[*F* ^2^ > 2σ(*F* ^2^)] = 0.039
*wR*(*F* ^2^) = 0.104
*S* = 1.02
11260 reflections
780 parameters
20 restraints
H atoms treated by a mixture of independent and constrained refinement
Δρ_max_ = 0.75 e Å^−3^
Δρ_min_ = −0.28 e Å^−3^

### 

Data collection: *APEX2* (Bruker, 2007 ▶); cell refinement: *SAINT* (Bruker, 2007 ▶); data reduction: *SAINT*; program(s) used to solve structure: *SHELXS97* (Sheldrick, 2008 ▶); program(s) used to refine structure: *SHELXL97* (Sheldrick, 2008 ▶); molecular graphics: *X-SEED* (Barbour, 2001 ▶); software used to prepare material for publication: *SHELXL97* and *publCIF* (Westrip, 2010 ▶).

## Supplementary Material

Crystal structure: contains datablock(s) I, global. DOI: 10.1107/S1600536813001943/is5239sup1.cif

Structure factors: contains datablock(s) I. DOI: 10.1107/S1600536813001943/is5239Isup2.hkl

Supplementary material file. DOI: 10.1107/S1600536813001943/is5239Isup3.cml

Additional supplementary materials:  crystallographic information; 3D view; checkCIF report

## Figures and Tables

**Table 1:** Hydrogen-bond geometry (Å, °)

*D*—H⋯*A*	*D*—H	H⋯*A*	*D*⋯*A*	*D*—H⋯*A*
O2*A*—H2*A*⋯O5	0.81 (2)	1.86 (2)	2.6569 (16)	166 (2)
O2*B*—H2*B*⋯O1*B* ^i^	0.84 (1)	1.89 (2)	2.7111 (14)	165 (2)
O2*C*—H2*C*⋯O1*C* ^ii^	0.85 (1)	1.89 (2)	2.7089 (14)	162 (2)
O2*D*—H2*D*⋯O8^iii^	0.82 (2)	1.92 (2)	2.7134 (17)	164 (2)
O5—H5*E*⋯O1*C* ^ii^	0.86 (1)	1.94 (2)	2.7857 (15)	168 (2)
O5—H5*F*⋯O2*D* ^iii^	0.86 (1)	1.79 (1)	2.6490 (17)	178 (2)
O6—H6*F*⋯O2*C* ^iv^	0.90 (1)	1.99 (1)	2.8837 (16)	177 (2)
O6—H6*E*⋯O1*D* ^iii^	0.88 (1)	1.96 (2)	2.8218 (15)	167 (2)
O7—H7*F*⋯O1*A*	0.87 (1)	1.97 (2)	2.8399 (16)	176 (2)
O7—H7*E*⋯O2*B* ^v^	0.89 (1)	2.01 (1)	2.8987 (16)	175 (2)
O8—H8*E*⋯O7	0.95 (2)	1.87 (2)	2.7637 (18)	156 (2)
O8—H8*F*⋯O9	0.91 (1)	1.93 (2)	2.8193 (18)	168 (2)
O9—H9*F*⋯O2*A*	0.84 (2)	1.98 (2)	2.8199 (17)	176 (2)
O9—H9*E*⋯O6	0.90 (1)	1.93 (2)	2.7724 (18)	155 (2)
C3*B*—H3*B*⋯O1*A* ^vi^	1.00	2.45	3.2581 (17)	138
C3*C*—H3*C*⋯O1*D*	1.00	2.31	3.1537 (17)	142
C3*D*—H3*D*⋯O1*D* ^iii^	1.00	2.56	3.4252 (18)	145
C5*A*—H5*A*⋯O7^v^	1.00	2.34	3.2374 (18)	148
C5*B*—H5*B*⋯O9^vi^	1.00	2.40	3.2568 (19)	143
C5*C*—H5*C*⋯O8^iii^	1.00	2.49	3.2395 (19)	131
C5*D*—H5*D*⋯O6	1.00	2.41	3.2010 (18)	135
C7*A*—H7*A*⋯O3*B*	0.95	2.38	3.2943 (17)	162
C7*B*—H7*B*⋯O3*A* ^vi^	0.95	2.46	3.3253 (18)	151
C7*C*—H7*C*⋯O3*D*	0.95	2.39	3.2704 (18)	155
C11*A*—H11*A*⋯O4*B* ^iv^	0.95	2.54	3.3027 (17)	138

Symmetry codes: (i) 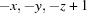; (ii) 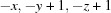; (iii) 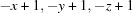; (iv) 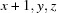; (v) 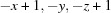; (vi) 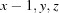.

**Fig. 1.**
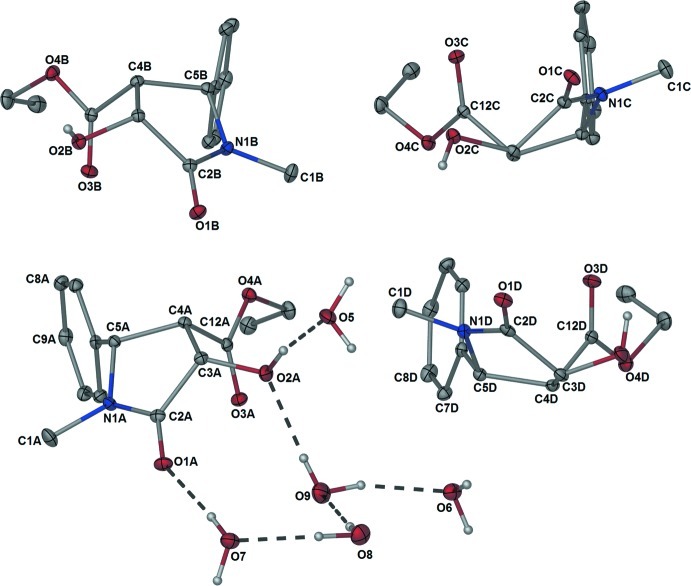
The asymmetric unit of the title compound, showing thermal ellipsoids at the 30% prbability level. C-bound hydrogen atoms are omitted for clarity.

**Fig. 2.**
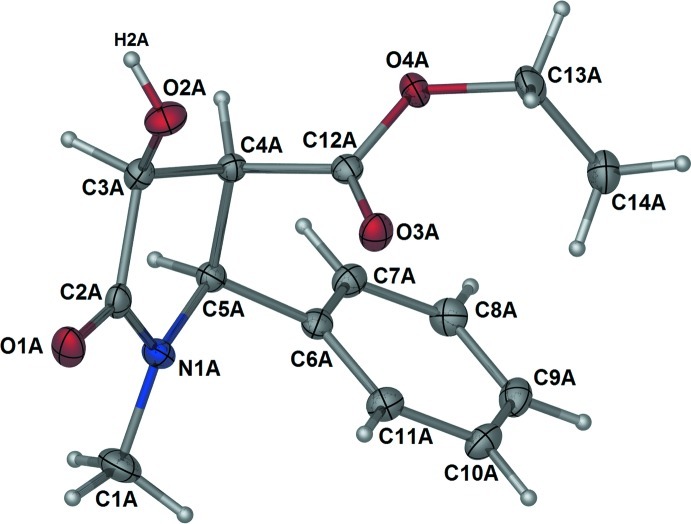
The molecular structure of one of the four independent organic molecules in the asymmetric unit (molecule A), showing atom-labelling scheme and thermal ellipsoids at 50% probability levels. The other three organic molecules are labelled in a similar manner.

## supplementary crystallographic information

### Comment

Polyfunctionalized pyrrolidones have received significant attention in the past decades due to their role as intermediates for synthesizing more complex biologically important molecules (Ma & Jiang, 1998). The present pyrrolidone molecule was prepared through catalytic hydrogenation of the previously reported substituted 2,5-dihydropyrrole-2-one (Mohammat *et al.*, 2009). The asymmetric unit of the crystal (Fig. 1) contains four geometrically slightly different pyrrolidone molecules: A (Fig. 2), B, C & D. The weighted r.m.s. fit for the superposition of the non-H atoms is 0.097 Å (in A & B with inversion), 0.082 Å (in A & C), 0.107 Å (in A & D with inversion), 0.141 Å (in B & C with inversion), 0.034 Å (in B & D) and 0.138 Å (in C & D with inversion). The planes of the phenyl and pyrrolidine rings in the four molecules make dihedral angles of 60.87 (5), 64.45 (5), 62.03 (5) and 65.79 (5)°. The bond lengths and angles in the four molecules are comparable to those in similar structures (Chu *et al.*, 2011; Gainsford & Mason, 2010). There are five crystallographically independent water molecules in the crystal structure, connecting the pyrrolidone molecules into a layer parallel to the *ab* plane *via* O—H···O hydrogen bonds (Table 1). The supramolecular structure is further consolidated by intermolecular C—H···O interactions (Table 1).

### Experimental

The synthetic approach to the title compound began with the preparation of ethyl 4-hydroxy-1-methyl-5-oxo-2-phenyl-2,5-dihydropyrrole-3-carboxylate as reported by (Mohammat *et al.*, 2009). This compound was catalytically hydrogenated by addition of Pd—C (10% wt) (0.91 g, 8.60 mmol) to its stirring solution (1.69 g, 6.47 mmol) in ethanol (110 ml) under hydrogen atmosphere for 24 h. The mixture was then filtered through celite. After removal of the solvent, the crude product was purified by column chromatography using silica gel (eluent: EtOAc) to give the diastereomeric product as a white solid (1.20 g, 71%). Slow evaporation of an ethyl acetate solution of the product at room temperature gave colorless crystals of the title compound.

### Refinement

The C-bound hydrogen atoms were located in calculated positions and refined in a riding mode with C—H distances of 0.95–1.00 Å. The O-bound H atoms were found in a difference Fourier map and refined with distance restraints of O—H = 0.88 (2) and H···H = 1.41 (1) Å (for the water molecules) and O—H = 0.84 (2) Å (for the hydroxyl groups). For all hydrogen atoms, *U*_iso_ were set to 1.2–1.5*U*_eq_(carrier atom). The most disagreeable reflections with delta(*F^2^*)/e.s.d. >10 were omitted (4 reflections).

### Crystal data

**Table:**

C_14_H_17_NO_4_·1.25H_2_O	*Z* = 8
*M**_r_* = 285.81	*F*(000) = 1220
Triclinic, *P*1	*D*_x_ = 1.310 Mg m^−^^3^
Hall symbol: -P 1	Mo *K*α radiation, λ = 0.71073 Å
*a* = 10.3669 (2) Å	Cell parameters from 9907 reflections
*b* = 15.7466 (3) Å	θ = 2.4–29.7°
*c* = 18.4539 (3) Å	µ = 0.10 mm^−^^1^
α = 77.373 (1)°	*T* = 100 K
β = 80.425 (1)°	Block, colorless
γ = 87.058 (1)°	0.21 × 0.15 × 0.09 mm
*V* = 2898.31 (9) Å^3^	

### Data collection

**Table:**

Bruker APEXII CCD diffractometer	11260 independent reflections
Radiation source: fine-focus sealed tube	9637 reflections with *I* > 2σ(*I*)
Graphite monochromator	*R*_int_ = 0.018
φ and ω scans	θ_max_ = 26.0°, θ_min_ = 1.9°
Absorption correction: multi-scan (*SADABS*; Sheldrick, 1996)	*h* = −12→12
*T*_min_ = 0.979, *T*_max_ = 0.991	*k* = −19→19
22505 measured reflections	*l* = −22→22

### Refinement

**Table:**

Refinement on *F*^2^	Primary atom site location: structure-invariant direct methods
Least-squares matrix: full	Secondary atom site location: difference Fourier map
*R*[*F*^2^ > 2σ(*F*^2^)] = 0.039	Hydrogen site location: inferred from neighbouring sites
*wR*(*F*^2^) = 0.104	H atoms treated by a mixture of independent and constrained refinement
*S* = 1.02	*w* = 1/[σ^2^(*F*_o_^2^) + (0.0505*P*)^2^ + 1.2863*P*] where *P* = (*F*_o_^2^ + 2*F*_c_^2^)/3
11260 reflections	(Δ/σ)_max_ = 0.001
780 parameters	Δρ_max_ = 0.75 e Å^−^^3^
20 restraints	Δρ_min_ = −0.28 e Å^−^^3^

### Special details

**Table:**

Geometry. All e.s.d.'s (except the e.s.d. in the dihedral angle between two l.s. planes) are estimated using the full covariance matrix. The cell e.s.d.'s are taken into account individually in the estimation of e.s.d.'s in distances, angles and torsion angles; correlations between e.s.d.'s in cell parameters are only used when they are defined by crystal symmetry. An approximate (isotropic) treatment of cell e.s.d.'s is used for estimating e.s.d.'s involving l.s. planes.
Refinement. Refinement of *F*^2^ against ALL reflections. The weighted *R*-factor *wR* and goodness of fit *S* are based on *F*^2^, conventional *R*-factors *R* are based on *F*, with *F* set to zero for negative *F*^2^. The threshold expression of *F*^2^ > σ(*F*^2^) is used only for calculating *R*-factors(gt) *etc*. and is not relevant to the choice of reflections for refinement. *R*-factors based on *F*^2^ are statistically about twice as large as those based on *F*, and *R*- factors based on ALL data will be even larger.

### Fractional atomic coordinates and isotropic or equivalent isotropic displacement parameters (Å^2^)

**Table:**

	*x*	*y*	*z*	*U*_iso_*/*U*_eq_	
O1A	0.64901 (10)	0.04071 (7)	0.45502 (6)	0.0238 (2)	
O2A	0.49840 (11)	0.20181 (7)	0.41628 (6)	0.0242 (2)	
H2A	0.4372 (17)	0.2196 (13)	0.4425 (10)	0.036*	
O3A	0.64351 (10)	0.21091 (7)	0.26500 (6)	0.0220 (2)	
O4A	0.45909 (10)	0.25338 (6)	0.21664 (6)	0.0202 (2)	
N1A	0.58255 (11)	0.01013 (8)	0.35122 (7)	0.0188 (3)	
C1A	0.64186 (17)	−0.07648 (10)	0.35670 (10)	0.0303 (4)	
H1AA	0.6888	−0.0900	0.3999	0.045*	
H1AB	0.7033	−0.0783	0.3105	0.045*	
H1AC	0.5734	−0.1194	0.3634	0.045*	
C2A	0.57953 (13)	0.05509 (9)	0.40585 (8)	0.0180 (3)	
C3A	0.47030 (13)	0.12313 (9)	0.39841 (8)	0.0174 (3)	
H3A	0.3904	0.0985	0.4334	0.021*	
C4A	0.44481 (13)	0.13153 (9)	0.31760 (8)	0.0157 (3)	
H4A	0.3501	0.1440	0.3144	0.019*	
C5A	0.48550 (13)	0.04019 (9)	0.30133 (8)	0.0167 (3)	
H5A	0.4080	0.0013	0.3183	0.020*	
C6A	0.53553 (14)	0.04127 (9)	0.21912 (8)	0.0171 (3)	
C7A	0.44476 (14)	0.03707 (9)	0.17244 (8)	0.0200 (3)	
H7A	0.3546	0.0307	0.1929	0.024*	
C8A	0.48543 (15)	0.04213 (10)	0.09585 (9)	0.0228 (3)	
H8A	0.4227	0.0400	0.0642	0.027*	
C9A	0.61664 (15)	0.05028 (10)	0.06559 (9)	0.0230 (3)	
H9A	0.6442	0.0536	0.0133	0.028*	
C10A	0.70755 (14)	0.05353 (10)	0.11199 (9)	0.0228 (3)	
H10A	0.7979	0.0582	0.0916	0.027*	
C11A	0.66731 (14)	0.04995 (9)	0.18827 (8)	0.0205 (3)	
H11A	0.7301	0.0534	0.2195	0.025*	
C12A	0.52894 (13)	0.20185 (9)	0.26489 (8)	0.0164 (3)	
C13A	0.53044 (16)	0.32175 (10)	0.16058 (9)	0.0250 (3)	
H13C	0.5960	0.3461	0.1834	0.030*	
H13D	0.4689	0.3693	0.1437	0.030*	
C14A	0.59860 (16)	0.28733 (10)	0.09366 (9)	0.0270 (3)	
H14D	0.6651	0.2439	0.1096	0.040*	
H14E	0.6405	0.3354	0.0553	0.040*	
H14F	0.5344	0.2603	0.0727	0.040*	
O1B	0.13260 (10)	0.11970 (7)	0.45539 (6)	0.0224 (2)	
O2B	−0.00506 (10)	−0.01771 (7)	0.41779 (6)	0.0200 (2)	
H2B	−0.0561 (16)	−0.0427 (12)	0.4561 (9)	0.030*	
O3B	0.14893 (9)	0.03902 (7)	0.27056 (6)	0.0219 (2)	
O4B	−0.02826 (10)	0.01861 (7)	0.22088 (6)	0.0218 (2)	
N1B	0.07124 (11)	0.20140 (8)	0.34704 (7)	0.0178 (2)	
C1B	0.12896 (16)	0.28289 (10)	0.34959 (9)	0.0259 (3)	
H1BA	0.0596	0.3267	0.3554	0.039*	
H1BB	0.1894	0.3032	0.3028	0.039*	
H1BC	0.1767	0.2736	0.3923	0.039*	
C2B	0.06659 (13)	0.13149 (9)	0.40419 (8)	0.0172 (3)	
C3B	−0.03891 (13)	0.07115 (9)	0.39767 (8)	0.0167 (3)	
H3B	−0.1214	0.0828	0.4309	0.020*	
C4B	−0.05891 (13)	0.10050 (9)	0.31525 (8)	0.0163 (3)	
H4B	−0.1517	0.0927	0.3098	0.020*	
C5B	−0.02377 (13)	0.19877 (9)	0.29672 (8)	0.0167 (3)	
H5B	−0.1037	0.2327	0.3126	0.020*	
C6B	0.02584 (14)	0.23547 (9)	0.21441 (8)	0.0169 (3)	
C7B	−0.06444 (14)	0.27310 (10)	0.16819 (8)	0.0208 (3)	
H7B	−0.1540	0.2775	0.1891	0.025*	
C8B	−0.02433 (15)	0.30423 (10)	0.09168 (9)	0.0244 (3)	
H8B	−0.0866	0.3294	0.0605	0.029*	
C9B	0.10633 (16)	0.29868 (10)	0.06066 (9)	0.0246 (3)	
H9B	0.1336	0.3199	0.0083	0.029*	
C10B	0.19687 (15)	0.26215 (10)	0.10625 (9)	0.0227 (3)	
H10B	0.2866	0.2587	0.0851	0.027*	
C11B	0.15721 (14)	0.23044 (9)	0.18279 (8)	0.0200 (3)	
H11B	0.2199	0.2052	0.2137	0.024*	
C12B	0.03408 (13)	0.04982 (9)	0.26770 (8)	0.0165 (3)	
C13B	0.05179 (17)	−0.02891 (10)	0.16992 (9)	0.0288 (4)	
H13E	−0.0025	−0.0723	0.1579	0.035*	
H13F	0.1231	−0.0605	0.1948	0.035*	
C14B	0.10956 (16)	0.03211 (11)	0.09840 (9)	0.0296 (4)	
H14G	0.0393	0.0663	0.0756	0.044*	
H14H	0.1568	−0.0016	0.0633	0.044*	
H14I	0.1701	0.0713	0.1098	0.044*	
O1C	−0.10524 (10)	0.63323 (7)	0.46461 (6)	0.0221 (2)	
O2C	0.03047 (10)	0.48233 (6)	0.41740 (6)	0.0204 (2)	
H2C	0.0678 (17)	0.4530 (11)	0.4525 (9)	0.031*	
O3C	−0.04204 (10)	0.54456 (7)	0.26963 (6)	0.0211 (2)	
O4C	0.16347 (10)	0.51174 (7)	0.22014 (6)	0.0211 (2)	
N1C	0.00350 (11)	0.70649 (8)	0.35085 (7)	0.0177 (2)	
C1C	−0.04334 (16)	0.79432 (10)	0.35577 (9)	0.0259 (3)	
H1CA	−0.0986	0.7928	0.4046	0.039*	
H1CB	−0.0944	0.8173	0.3154	0.039*	
H1CC	0.0315	0.8319	0.3506	0.039*	
C2C	−0.01997 (13)	0.63793 (9)	0.40823 (8)	0.0173 (3)	
C3C	0.08167 (13)	0.56697 (9)	0.39700 (8)	0.0166 (3)	
H3C	0.1509	0.5699	0.4282	0.020*	
C4C	0.14199 (13)	0.59354 (9)	0.31376 (8)	0.0160 (3)	
H4C	0.2379	0.5798	0.3068	0.019*	
C5C	0.11761 (13)	0.69378 (9)	0.29505 (8)	0.0162 (3)	
H5C	0.1942	0.7229	0.3052	0.019*	
C6C	0.09907 (13)	0.73012 (9)	0.21423 (8)	0.0168 (3)	
C7C	0.21080 (14)	0.75141 (9)	0.16050 (8)	0.0199 (3)	
H7C	0.2946	0.7460	0.1757	0.024*	
C8C	0.20017 (15)	0.78040 (10)	0.08495 (8)	0.0237 (3)	
H8C	0.2767	0.7940	0.0486	0.028*	
C9C	0.07817 (16)	0.78955 (10)	0.06253 (9)	0.0254 (3)	
H9C	0.0708	0.8099	0.0110	0.030*	
C10C	−0.03285 (15)	0.76886 (10)	0.11569 (9)	0.0243 (3)	
H10C	−0.1166	0.7752	0.1005	0.029*	
C11C	−0.02266 (14)	0.73883 (9)	0.19119 (8)	0.0205 (3)	
H11C	−0.0993	0.7242	0.2272	0.025*	
C12C	0.07442 (13)	0.54789 (9)	0.26684 (8)	0.0165 (3)	
C13C	0.11189 (16)	0.47051 (10)	0.16792 (9)	0.0262 (3)	
H13G	0.0291	0.4411	0.1931	0.031*	
H13H	0.1751	0.4259	0.1527	0.031*	
C14C	0.08725 (16)	0.53705 (11)	0.09926 (9)	0.0269 (3)	
H14J	0.0219	0.5798	0.1142	0.040*	
H14K	0.0548	0.5080	0.0641	0.040*	
H14L	0.1690	0.5666	0.0748	0.040*	
O1D	0.35787 (10)	0.51833 (7)	0.44621 (6)	0.0237 (2)	
O2D	0.50430 (11)	0.67992 (7)	0.41422 (7)	0.0290 (3)	
H2D	0.4278 (16)	0.6933 (14)	0.4118 (12)	0.043*	
O3D	0.44459 (10)	0.69891 (7)	0.26203 (6)	0.0256 (2)	
O4D	0.64862 (10)	0.74790 (7)	0.21751 (6)	0.0235 (2)	
N1D	0.48531 (11)	0.49696 (8)	0.33763 (7)	0.0177 (2)	
C1D	0.43039 (16)	0.41419 (10)	0.33667 (9)	0.0262 (3)	
H1DA	0.3635	0.3972	0.3812	0.039*	
H1DB	0.4999	0.3696	0.3371	0.039*	
H1DC	0.3907	0.4200	0.2912	0.039*	
C2D	0.45232 (14)	0.53649 (9)	0.39610 (8)	0.0185 (3)	
C3D	0.55444 (14)	0.60389 (9)	0.39124 (8)	0.0194 (3)	
H3D	0.6183	0.5768	0.4250	0.023*	
C4D	0.62603 (13)	0.61722 (9)	0.30972 (8)	0.0174 (3)	
H4D	0.7209	0.6288	0.3068	0.021*	
C5D	0.60730 (13)	0.52840 (9)	0.28869 (8)	0.0169 (3)	
H5D	0.6801	0.4880	0.3042	0.020*	
C6D	0.60521 (14)	0.53365 (9)	0.20598 (8)	0.0173 (3)	
C7D	0.72226 (14)	0.51983 (10)	0.16043 (8)	0.0211 (3)	
H7D	0.7999	0.5058	0.1819	0.025*	
C8D	0.72600 (16)	0.52651 (11)	0.08379 (9)	0.0261 (3)	
H8D	0.8062	0.5169	0.0531	0.031*	
C9D	0.61378 (16)	0.54704 (10)	0.05188 (9)	0.0265 (3)	
H9D	0.6168	0.5521	−0.0006	0.032*	
C10D	0.49682 (16)	0.56017 (10)	0.09706 (9)	0.0247 (3)	
H10D	0.4193	0.5739	0.0754	0.030*	
C11D	0.49217 (14)	0.55343 (9)	0.17381 (8)	0.0205 (3)	
H11D	0.4116	0.5623	0.2044	0.025*	
C12D	0.56095 (14)	0.69130 (9)	0.26130 (8)	0.0187 (3)	
C13D	0.59552 (17)	0.82097 (10)	0.16744 (10)	0.0300 (4)	
H13A	0.6548	0.8711	0.1567	0.036*	
H13B	0.5094	0.8386	0.1924	0.036*	
C14D	0.58004 (17)	0.79696 (11)	0.09471 (9)	0.0313 (4)	
H14A	0.6652	0.7792	0.0701	0.047*	
H14B	0.5458	0.8473	0.0615	0.047*	
H14C	0.5190	0.7487	0.1052	0.047*	
O5	0.32498 (11)	0.25906 (7)	0.51861 (8)	0.0321 (3)	
H5E	0.2567 (15)	0.2925 (13)	0.5169 (12)	0.048*	
H5F	0.3790 (16)	0.2792 (13)	0.5414 (12)	0.048*	
O6	0.76502 (11)	0.43383 (8)	0.41992 (6)	0.0287 (3)	
H6E	0.7240 (16)	0.4400 (13)	0.4641 (9)	0.043*	
H6F	0.8469 (14)	0.4507 (13)	0.4182 (11)	0.043*	
O7	0.73659 (11)	0.07895 (8)	0.58207 (7)	0.0302 (3)	
H7E	0.8181 (15)	0.0577 (13)	0.5805 (11)	0.045*	
H7F	0.7098 (17)	0.0702 (14)	0.5420 (10)	0.045*	
O8	0.74438 (13)	0.25573 (9)	0.57526 (7)	0.0376 (3)	
H8E	0.732 (2)	0.1948 (10)	0.5919 (10)	0.056*	
H8F	0.758 (2)	0.2590 (13)	0.5249 (8)	0.056*	
O9	0.74986 (13)	0.25813 (9)	0.42157 (7)	0.0378 (3)	
H9E	0.747 (2)	0.3165 (10)	0.4075 (12)	0.057*	
H9F	0.6757 (17)	0.2394 (12)	0.4216 (13)	0.057*	

### Atomic displacement parameters (Å^2^)

**Table:**

	*U*^11^	*U*^22^	*U*^33^	*U*^12^	*U*^13^	*U*^23^
O1A	0.0207 (5)	0.0312 (6)	0.0185 (5)	−0.0011 (4)	−0.0069 (4)	0.0002 (4)
O2A	0.0252 (6)	0.0248 (6)	0.0255 (6)	0.0023 (4)	−0.0021 (5)	−0.0139 (5)
O3A	0.0175 (5)	0.0255 (6)	0.0218 (5)	−0.0040 (4)	−0.0019 (4)	−0.0027 (4)
O4A	0.0223 (5)	0.0180 (5)	0.0183 (5)	0.0004 (4)	−0.0038 (4)	0.0008 (4)
N1A	0.0196 (6)	0.0174 (6)	0.0189 (6)	0.0035 (5)	−0.0047 (5)	−0.0024 (5)
C1A	0.0361 (9)	0.0210 (8)	0.0326 (9)	0.0100 (7)	−0.0086 (7)	−0.0033 (7)
C2A	0.0153 (7)	0.0207 (7)	0.0153 (7)	−0.0033 (5)	0.0001 (5)	0.0007 (6)
C3A	0.0161 (7)	0.0204 (7)	0.0154 (7)	−0.0009 (5)	−0.0008 (5)	−0.0043 (6)
C4A	0.0135 (6)	0.0174 (7)	0.0157 (7)	0.0008 (5)	−0.0019 (5)	−0.0030 (5)
C5A	0.0155 (6)	0.0162 (7)	0.0176 (7)	−0.0004 (5)	−0.0022 (5)	−0.0025 (6)
C6A	0.0189 (7)	0.0145 (7)	0.0179 (7)	−0.0003 (5)	−0.0018 (6)	−0.0042 (5)
C7A	0.0176 (7)	0.0208 (7)	0.0217 (8)	−0.0017 (5)	−0.0011 (6)	−0.0062 (6)
C8A	0.0245 (8)	0.0248 (8)	0.0213 (8)	−0.0006 (6)	−0.0062 (6)	−0.0079 (6)
C9A	0.0298 (8)	0.0220 (7)	0.0170 (7)	−0.0023 (6)	0.0007 (6)	−0.0066 (6)
C10A	0.0182 (7)	0.0253 (8)	0.0246 (8)	−0.0032 (6)	0.0029 (6)	−0.0090 (6)
C11A	0.0183 (7)	0.0224 (7)	0.0225 (8)	−0.0007 (6)	−0.0035 (6)	−0.0078 (6)
C12A	0.0189 (7)	0.0153 (7)	0.0158 (7)	0.0012 (5)	−0.0023 (5)	−0.0059 (5)
C13A	0.0329 (8)	0.0168 (7)	0.0218 (8)	−0.0012 (6)	−0.0026 (6)	0.0021 (6)
C14A	0.0315 (8)	0.0271 (8)	0.0201 (8)	−0.0036 (7)	−0.0021 (6)	−0.0011 (6)
O1B	0.0234 (5)	0.0263 (6)	0.0171 (5)	−0.0056 (4)	−0.0067 (4)	−0.0002 (4)
O2B	0.0225 (5)	0.0178 (5)	0.0169 (5)	−0.0022 (4)	−0.0003 (4)	0.0003 (4)
O3B	0.0165 (5)	0.0271 (6)	0.0205 (5)	0.0028 (4)	−0.0012 (4)	−0.0037 (4)
O4B	0.0220 (5)	0.0245 (5)	0.0213 (5)	−0.0019 (4)	−0.0022 (4)	−0.0108 (4)
N1B	0.0197 (6)	0.0180 (6)	0.0159 (6)	−0.0034 (5)	−0.0039 (5)	−0.0029 (5)
C1B	0.0362 (9)	0.0202 (8)	0.0227 (8)	−0.0080 (6)	−0.0074 (7)	−0.0036 (6)
C2B	0.0164 (7)	0.0203 (7)	0.0138 (7)	−0.0007 (5)	0.0005 (5)	−0.0033 (6)
C3B	0.0158 (7)	0.0183 (7)	0.0154 (7)	−0.0008 (5)	−0.0008 (5)	−0.0032 (6)
C4B	0.0139 (6)	0.0194 (7)	0.0155 (7)	−0.0013 (5)	−0.0014 (5)	−0.0038 (6)
C5B	0.0160 (7)	0.0180 (7)	0.0162 (7)	0.0005 (5)	−0.0026 (5)	−0.0038 (6)
C6B	0.0199 (7)	0.0149 (7)	0.0163 (7)	−0.0024 (5)	−0.0027 (5)	−0.0038 (5)
C7B	0.0199 (7)	0.0218 (7)	0.0211 (7)	−0.0024 (6)	−0.0044 (6)	−0.0039 (6)
C8B	0.0285 (8)	0.0243 (8)	0.0210 (8)	−0.0039 (6)	−0.0103 (6)	−0.0008 (6)
C9B	0.0330 (8)	0.0244 (8)	0.0153 (7)	−0.0086 (6)	−0.0008 (6)	−0.0025 (6)
C10B	0.0212 (7)	0.0239 (8)	0.0222 (8)	−0.0047 (6)	0.0018 (6)	−0.0059 (6)
C11B	0.0202 (7)	0.0199 (7)	0.0199 (7)	−0.0022 (5)	−0.0037 (6)	−0.0037 (6)
C12B	0.0186 (7)	0.0152 (7)	0.0142 (7)	−0.0022 (5)	−0.0010 (5)	−0.0008 (5)
C13B	0.0365 (9)	0.0251 (8)	0.0271 (9)	0.0000 (7)	0.0002 (7)	−0.0150 (7)
C14B	0.0273 (8)	0.0375 (9)	0.0246 (8)	0.0030 (7)	−0.0005 (7)	−0.0116 (7)
O1C	0.0210 (5)	0.0224 (5)	0.0182 (5)	0.0060 (4)	0.0027 (4)	0.0003 (4)
O2C	0.0247 (5)	0.0174 (5)	0.0173 (5)	0.0016 (4)	−0.0045 (4)	0.0007 (4)
O3C	0.0181 (5)	0.0252 (5)	0.0200 (5)	−0.0024 (4)	−0.0034 (4)	−0.0040 (4)
O4C	0.0226 (5)	0.0230 (5)	0.0199 (5)	0.0032 (4)	−0.0035 (4)	−0.0102 (4)
N1C	0.0186 (6)	0.0170 (6)	0.0158 (6)	0.0025 (5)	0.0002 (5)	−0.0022 (5)
C1C	0.0316 (8)	0.0183 (7)	0.0235 (8)	0.0058 (6)	0.0023 (7)	−0.0015 (6)
C2C	0.0170 (7)	0.0192 (7)	0.0155 (7)	0.0014 (5)	−0.0039 (5)	−0.0029 (6)
C3C	0.0171 (7)	0.0173 (7)	0.0147 (7)	0.0019 (5)	−0.0032 (5)	−0.0023 (5)
C4C	0.0140 (6)	0.0180 (7)	0.0157 (7)	0.0020 (5)	−0.0024 (5)	−0.0034 (5)
C5C	0.0147 (6)	0.0176 (7)	0.0157 (7)	−0.0003 (5)	−0.0012 (5)	−0.0034 (5)
C6C	0.0191 (7)	0.0144 (7)	0.0172 (7)	−0.0004 (5)	−0.0032 (5)	−0.0037 (5)
C7C	0.0193 (7)	0.0204 (7)	0.0195 (7)	−0.0010 (5)	−0.0029 (6)	−0.0032 (6)
C8C	0.0278 (8)	0.0238 (8)	0.0173 (7)	−0.0029 (6)	0.0009 (6)	−0.0022 (6)
C9C	0.0385 (9)	0.0208 (7)	0.0176 (7)	−0.0015 (6)	−0.0103 (7)	−0.0011 (6)
C10C	0.0259 (8)	0.0229 (8)	0.0263 (8)	−0.0006 (6)	−0.0133 (6)	−0.0032 (6)
C11C	0.0192 (7)	0.0193 (7)	0.0224 (8)	−0.0016 (5)	−0.0036 (6)	−0.0026 (6)
C12C	0.0189 (7)	0.0151 (7)	0.0139 (7)	0.0009 (5)	−0.0025 (5)	0.0000 (5)
C13C	0.0336 (9)	0.0242 (8)	0.0247 (8)	0.0027 (6)	−0.0067 (7)	−0.0127 (7)
C14C	0.0287 (8)	0.0334 (9)	0.0204 (8)	−0.0030 (7)	−0.0030 (6)	−0.0093 (7)
O1D	0.0205 (5)	0.0320 (6)	0.0159 (5)	0.0039 (4)	0.0000 (4)	−0.0026 (4)
O2D	0.0264 (6)	0.0300 (6)	0.0358 (7)	0.0114 (5)	−0.0078 (5)	−0.0190 (5)
O3D	0.0186 (5)	0.0286 (6)	0.0276 (6)	0.0025 (4)	−0.0066 (4)	0.0000 (5)
O4D	0.0237 (5)	0.0190 (5)	0.0263 (6)	−0.0029 (4)	−0.0054 (4)	0.0000 (4)
N1D	0.0195 (6)	0.0185 (6)	0.0141 (6)	−0.0009 (5)	−0.0006 (5)	−0.0031 (5)
C1D	0.0320 (8)	0.0214 (8)	0.0230 (8)	−0.0070 (6)	0.0013 (7)	−0.0029 (6)
C2D	0.0183 (7)	0.0216 (7)	0.0145 (7)	0.0060 (5)	−0.0047 (6)	−0.0011 (6)
C3D	0.0199 (7)	0.0228 (7)	0.0176 (7)	0.0057 (6)	−0.0063 (6)	−0.0080 (6)
C4D	0.0155 (7)	0.0193 (7)	0.0182 (7)	0.0004 (5)	−0.0045 (5)	−0.0045 (6)
C5D	0.0166 (7)	0.0180 (7)	0.0156 (7)	0.0010 (5)	−0.0019 (5)	−0.0034 (5)
C6D	0.0204 (7)	0.0154 (7)	0.0154 (7)	−0.0024 (5)	−0.0012 (6)	−0.0029 (5)
C7D	0.0190 (7)	0.0245 (8)	0.0201 (7)	−0.0030 (6)	−0.0014 (6)	−0.0063 (6)
C8D	0.0255 (8)	0.0318 (9)	0.0197 (8)	−0.0075 (6)	0.0056 (6)	−0.0082 (7)
C9D	0.0372 (9)	0.0282 (8)	0.0144 (7)	−0.0099 (7)	−0.0021 (6)	−0.0044 (6)
C10D	0.0277 (8)	0.0259 (8)	0.0219 (8)	−0.0045 (6)	−0.0091 (6)	−0.0033 (6)
C11D	0.0203 (7)	0.0219 (7)	0.0189 (7)	−0.0018 (6)	−0.0017 (6)	−0.0044 (6)
C12D	0.0196 (7)	0.0184 (7)	0.0198 (7)	0.0000 (5)	−0.0046 (6)	−0.0065 (6)
C13D	0.0360 (9)	0.0175 (7)	0.0337 (9)	−0.0021 (6)	−0.0084 (7)	0.0032 (7)
C14D	0.0304 (9)	0.0313 (9)	0.0282 (9)	−0.0014 (7)	−0.0067 (7)	0.0039 (7)
O5	0.0199 (6)	0.0264 (6)	0.0495 (8)	0.0046 (5)	−0.0041 (5)	−0.0093 (5)
O6	0.0268 (6)	0.0369 (7)	0.0243 (6)	0.0008 (5)	−0.0031 (5)	−0.0117 (5)
O7	0.0267 (6)	0.0350 (7)	0.0300 (6)	0.0034 (5)	−0.0075 (5)	−0.0081 (5)
O8	0.0438 (7)	0.0417 (7)	0.0315 (7)	−0.0013 (6)	−0.0086 (6)	−0.0146 (6)
O9	0.0423 (7)	0.0394 (7)	0.0359 (7)	0.0054 (6)	−0.0062 (6)	−0.0188 (6)

### Geometric parameters (Å, º)

**Table:**

O1A—C2A	1.2266 (17)	O4C—C13C	1.4549 (18)
O2A—C3A	1.4057 (17)	N1C—C2C	1.3371 (18)
O2A—H2A	0.809 (15)	N1C—C1C	1.4589 (18)
O3A—C12A	1.2039 (17)	N1C—C5C	1.4698 (17)
O4A—C12A	1.3469 (17)	C1C—H1CA	0.9800
O4A—C13A	1.4548 (17)	C1C—H1CB	0.9800
N1A—C2A	1.3482 (19)	C1C—H1CC	0.9800
N1A—C1A	1.4568 (19)	C2C—C3C	1.5196 (19)
N1A—C5A	1.4671 (18)	C3C—C4C	1.5337 (19)
C1A—H1AA	0.9800	C3C—H3C	1.0000
C1A—H1AB	0.9800	C4C—C12C	1.5109 (19)
C1A—H1AC	0.9800	C4C—C5C	1.5571 (19)
C2A—C3A	1.5205 (19)	C4C—H4C	1.0000
C3A—C4A	1.5338 (19)	C5C—C6C	1.5164 (19)
C3A—H3A	1.0000	C5C—H5C	1.0000
C4A—C12A	1.5095 (19)	C6C—C11C	1.388 (2)
C4A—C5A	1.5561 (19)	C6C—C7C	1.396 (2)
C4A—H4A	1.0000	C7C—C8C	1.390 (2)
C5A—C6A	1.5163 (19)	C7C—H7C	0.9500
C5A—H5A	1.0000	C8C—C9C	1.386 (2)
C6A—C7A	1.390 (2)	C8C—H8C	0.9500
C6A—C11A	1.391 (2)	C9C—C10C	1.384 (2)
C7A—C8A	1.392 (2)	C9C—H9C	0.9500
C7A—H7A	0.9500	C10C—C11C	1.390 (2)
C8A—C9A	1.383 (2)	C10C—H10C	0.9500
C8A—H8A	0.9500	C11C—H11C	0.9500
C9A—C10A	1.384 (2)	C13C—C14C	1.506 (2)
C9A—H9A	0.9500	C13C—H13G	0.9900
C10A—C11A	1.390 (2)	C13C—H13H	0.9900
C10A—H10A	0.9500	C14C—H14J	0.9800
C11A—H11A	0.9500	C14C—H14K	0.9800
C13A—C14A	1.510 (2)	C14C—H14L	0.9800
C13A—H13C	0.9900	O1D—C2D	1.2278 (18)
C13A—H13D	0.9900	O2D—C3D	1.4029 (17)
C14A—H14D	0.9800	O2D—H2D	0.815 (15)
C14A—H14E	0.9800	O3D—C12D	1.2042 (17)
C14A—H14F	0.9800	O4D—C12D	1.3406 (18)
O1B—C2B	1.2333 (17)	O4D—C13D	1.4561 (18)
O2B—C3B	1.4087 (17)	N1D—C2D	1.3481 (19)
O2B—H2B	0.842 (14)	N1D—C1D	1.4535 (19)
O3B—C12B	1.2023 (17)	N1D—C5D	1.4659 (18)
O4B—C12B	1.3454 (17)	C1D—H1DA	0.9800
O4B—C13B	1.4551 (18)	C1D—H1DB	0.9800
N1B—C2B	1.3443 (18)	C1D—H1DC	0.9800
N1B—C1B	1.4566 (18)	C2D—C3D	1.516 (2)
N1B—C5B	1.4695 (18)	C3D—C4D	1.538 (2)
C1B—H1BA	0.9800	C3D—H3D	1.0000
C1B—H1BB	0.9800	C4D—C12D	1.5094 (19)
C1B—H1BC	0.9800	C4D—C5D	1.5603 (19)
C2B—C3B	1.5210 (19)	C4D—H4D	1.0000
C3B—C4B	1.5356 (19)	C5D—C6D	1.5142 (19)
C3B—H3B	1.0000	C5D—H5D	1.0000
C4B—C12B	1.5105 (19)	C6D—C11D	1.391 (2)
C4B—C5B	1.5587 (19)	C6D—C7D	1.393 (2)
C4B—H4B	1.0000	C7D—C8D	1.389 (2)
C5B—C6B	1.5142 (19)	C7D—H7D	0.9500
C5B—H5B	1.0000	C8D—C9D	1.382 (2)
C6B—C7B	1.394 (2)	C8D—H8D	0.9500
C6B—C11B	1.395 (2)	C9D—C10D	1.386 (2)
C7B—C8B	1.389 (2)	C9D—H9D	0.9500
C7B—H7B	0.9500	C10D—C11D	1.390 (2)
C8B—C9B	1.386 (2)	C10D—H10D	0.9500
C8B—H8B	0.9500	C11D—H11D	0.9500
C9B—C10B	1.384 (2)	C13D—C14D	1.506 (2)
C9B—H9B	0.9500	C13D—H13A	0.9900
C10B—C11B	1.390 (2)	C13D—H13B	0.9900
C10B—H10B	0.9500	C14D—H14A	0.9800
C11B—H11B	0.9500	C14D—H14B	0.9800
C13B—C14B	1.505 (2)	C14D—H14C	0.9800
C13B—H13E	0.9900	O5—H5E	0.861 (14)
C13B—H13F	0.9900	O5—H5F	0.864 (14)
C14B—H14G	0.9800	O6—H6E	0.877 (14)
C14B—H14H	0.9800	O6—H6F	0.895 (14)
C14B—H14I	0.9800	O7—H7E	0.892 (14)
O1C—C2C	1.2395 (17)	O7—H7F	0.871 (14)
O2C—C3C	1.4096 (17)	O8—H8E	0.949 (15)
O2C—H2C	0.847 (14)	O8—H8F	0.906 (14)
O3C—C12C	1.2031 (17)	O9—H9E	0.899 (14)
O4C—C12C	1.3472 (17)	O9—H9F	0.838 (15)
			
C3A—O2A—H2A	112.0 (14)	C2C—N1C—C5C	113.34 (11)
C12A—O4A—C13A	116.42 (11)	C1C—N1C—C5C	119.94 (11)
C2A—N1A—C1A	121.99 (13)	N1C—C1C—H1CA	109.5
C2A—N1A—C5A	113.88 (11)	N1C—C1C—H1CB	109.5
C1A—N1A—C5A	120.92 (12)	H1CA—C1C—H1CB	109.5
N1A—C1A—H1AA	109.5	N1C—C1C—H1CC	109.5
N1A—C1A—H1AB	109.5	H1CA—C1C—H1CC	109.5
H1AA—C1A—H1AB	109.5	H1CB—C1C—H1CC	109.5
N1A—C1A—H1AC	109.5	O1C—C2C—N1C	126.51 (13)
H1AA—C1A—H1AC	109.5	O1C—C2C—C3C	124.09 (12)
H1AB—C1A—H1AC	109.5	N1C—C2C—C3C	109.26 (12)
O1A—C2A—N1A	125.70 (13)	O2C—C3C—C2C	113.76 (11)
O1A—C2A—C3A	126.07 (13)	O2C—C3C—C4C	113.58 (11)
N1A—C2A—C3A	108.15 (12)	C2C—C3C—C4C	103.96 (11)
O2A—C3A—C2A	113.95 (11)	O2C—C3C—H3C	108.4
O2A—C3A—C4A	114.69 (11)	C2C—C3C—H3C	108.4
C2A—C3A—C4A	103.82 (11)	C4C—C3C—H3C	108.4
O2A—C3A—H3A	108.0	C12C—C4C—C3C	110.32 (11)
C2A—C3A—H3A	108.0	C12C—C4C—C5C	111.80 (11)
C4A—C3A—H3A	108.0	C3C—C4C—C5C	103.19 (11)
C12A—C4A—C3A	110.07 (11)	C12C—C4C—H4C	110.4
C12A—C4A—C5A	111.26 (11)	C3C—C4C—H4C	110.4
C3A—C4A—C5A	103.00 (11)	C5C—C4C—H4C	110.4
C12A—C4A—H4A	110.8	N1C—C5C—C6C	114.00 (11)
C3A—C4A—H4A	110.8	N1C—C5C—C4C	103.28 (10)
C5A—C4A—H4A	110.8	C6C—C5C—C4C	113.98 (11)
N1A—C5A—C6A	114.11 (11)	N1C—C5C—H5C	108.4
N1A—C5A—C4A	102.66 (11)	C6C—C5C—H5C	108.4
C6A—C5A—C4A	113.89 (11)	C4C—C5C—H5C	108.4
N1A—C5A—H5A	108.6	C11C—C6C—C7C	119.09 (13)
C6A—C5A—H5A	108.6	C11C—C6C—C5C	122.96 (13)
C4A—C5A—H5A	108.6	C7C—C6C—C5C	117.87 (12)
C7A—C6A—C11A	119.07 (13)	C8C—C7C—C6C	120.38 (14)
C7A—C6A—C5A	118.23 (12)	C8C—C7C—H7C	119.8
C11A—C6A—C5A	122.65 (13)	C6C—C7C—H7C	119.8
C6A—C7A—C8A	120.30 (13)	C9C—C8C—C7C	120.14 (14)
C6A—C7A—H7A	119.9	C9C—C8C—H8C	119.9
C8A—C7A—H7A	119.9	C7C—C8C—H8C	119.9
C9A—C8A—C7A	120.36 (14)	C10C—C9C—C8C	119.63 (14)
C9A—C8A—H8A	119.8	C10C—C9C—H9C	120.2
C7A—C8A—H8A	119.8	C8C—C9C—H9C	120.2
C8A—C9A—C10A	119.55 (14)	C9C—C10C—C11C	120.46 (14)
C8A—C9A—H9A	120.2	C9C—C10C—H10C	119.8
C10A—C9A—H9A	120.2	C11C—C10C—H10C	119.8
C9A—C10A—C11A	120.35 (14)	C6C—C11C—C10C	120.29 (14)
C9A—C10A—H10A	119.8	C6C—C11C—H11C	119.9
C11A—C10A—H10A	119.8	C10C—C11C—H11C	119.9
C10A—C11A—C6A	120.36 (14)	O3C—C12C—O4C	124.24 (13)
C10A—C11A—H11A	119.8	O3C—C12C—C4C	125.48 (13)
C6A—C11A—H11A	119.8	O4C—C12C—C4C	110.28 (11)
O3A—C12A—O4A	123.95 (13)	O4C—C13C—C14C	110.57 (12)
O3A—C12A—C4A	125.07 (13)	O4C—C13C—H13G	109.5
O4A—C12A—C4A	110.98 (11)	C14C—C13C—H13G	109.5
O4A—C13A—C14A	111.17 (12)	O4C—C13C—H13H	109.5
O4A—C13A—H13C	109.4	C14C—C13C—H13H	109.5
C14A—C13A—H13C	109.4	H13G—C13C—H13H	108.1
O4A—C13A—H13D	109.4	C13C—C14C—H14J	109.5
C14A—C13A—H13D	109.4	C13C—C14C—H14K	109.5
H13C—C13A—H13D	108.0	H14J—C14C—H14K	109.5
C13A—C14A—H14D	109.5	C13C—C14C—H14L	109.5
C13A—C14A—H14E	109.5	H14J—C14C—H14L	109.5
H14D—C14A—H14E	109.5	H14K—C14C—H14L	109.5
C13A—C14A—H14F	109.5	C3D—O2D—H2D	118.6 (15)
H14D—C14A—H14F	109.5	C12D—O4D—C13D	115.86 (12)
H14E—C14A—H14F	109.5	C2D—N1D—C1D	122.12 (12)
C3B—O2B—H2B	110.7 (13)	C2D—N1D—C5D	113.71 (12)
C12B—O4B—C13B	116.45 (11)	C1D—N1D—C5D	121.43 (12)
C2B—N1B—C1B	122.08 (12)	N1D—C1D—H1DA	109.5
C2B—N1B—C5B	113.31 (11)	N1D—C1D—H1DB	109.5
C1B—N1B—C5B	121.34 (12)	H1DA—C1D—H1DB	109.5
N1B—C1B—H1BA	109.5	N1D—C1D—H1DC	109.5
N1B—C1B—H1BB	109.5	H1DA—C1D—H1DC	109.5
H1BA—C1B—H1BB	109.5	H1DB—C1D—H1DC	109.5
N1B—C1B—H1BC	109.5	O1D—C2D—N1D	125.38 (14)
H1BA—C1B—H1BC	109.5	O1D—C2D—C3D	126.01 (13)
H1BB—C1B—H1BC	109.5	N1D—C2D—C3D	108.56 (12)
O1B—C2B—N1B	125.88 (13)	O2D—C3D—C2D	114.35 (12)
O1B—C2B—C3B	125.36 (13)	O2D—C3D—C4D	115.37 (12)
N1B—C2B—C3B	108.68 (12)	C2D—C3D—C4D	104.06 (11)
O2B—C3B—C2B	113.30 (11)	O2D—C3D—H3D	107.6
O2B—C3B—C4B	113.41 (11)	C2D—C3D—H3D	107.6
C2B—C3B—C4B	103.35 (11)	C4D—C3D—H3D	107.6
O2B—C3B—H3B	108.9	C12D—C4D—C3D	109.09 (11)
C2B—C3B—H3B	108.9	C12D—C4D—C5D	111.68 (11)
C4B—C3B—H3B	108.9	C3D—C4D—C5D	102.85 (11)
C12B—C4B—C3B	109.14 (11)	C12D—C4D—H4D	111.0
C12B—C4B—C5B	111.61 (11)	C3D—C4D—H4D	111.0
C3B—C4B—C5B	102.60 (11)	C5D—C4D—H4D	111.0
C12B—C4B—H4B	111.1	N1D—C5D—C6D	113.85 (11)
C3B—C4B—H4B	111.1	N1D—C5D—C4D	102.67 (11)
C5B—C4B—H4B	111.1	C6D—C5D—C4D	114.83 (11)
N1B—C5B—C6B	114.30 (11)	N1D—C5D—H5D	108.4
N1B—C5B—C4B	102.50 (11)	C6D—C5D—H5D	108.4
C6B—C5B—C4B	114.38 (11)	C4D—C5D—H5D	108.4
N1B—C5B—H5B	108.4	C11D—C6D—C7D	119.13 (13)
C6B—C5B—H5B	108.4	C11D—C6D—C5D	122.66 (13)
C4B—C5B—H5B	108.4	C7D—C6D—C5D	118.20 (13)
C7B—C6B—C11B	118.98 (13)	C8D—C7D—C6D	120.30 (14)
C7B—C6B—C5B	118.40 (12)	C8D—C7D—H7D	119.9
C11B—C6B—C5B	122.58 (13)	C6D—C7D—H7D	119.9
C8B—C7B—C6B	120.45 (14)	C9D—C8D—C7D	120.42 (14)
C8B—C7B—H7B	119.8	C9D—C8D—H8D	119.8
C6B—C7B—H7B	119.8	C7D—C8D—H8D	119.8
C9B—C8B—C7B	120.20 (14)	C8D—C9D—C10D	119.51 (14)
C9B—C8B—H8B	119.9	C8D—C9D—H9D	120.2
C7B—C8B—H8B	119.9	C10D—C9D—H9D	120.2
C10B—C9B—C8B	119.75 (14)	C9D—C10D—C11D	120.43 (14)
C10B—C9B—H9B	120.1	C9D—C10D—H10D	119.8
C8B—C9B—H9B	120.1	C11D—C10D—H10D	119.8
C9B—C10B—C11B	120.32 (14)	C10D—C11D—C6D	120.20 (14)
C9B—C10B—H10B	119.8	C10D—C11D—H11D	119.9
C11B—C10B—H10B	119.8	C6D—C11D—H11D	119.9
C10B—C11B—C6B	120.30 (14)	O3D—C12D—O4D	124.07 (13)
C10B—C11B—H11B	119.9	O3D—C12D—C4D	124.25 (13)
C6B—C11B—H11B	119.9	O4D—C12D—C4D	111.68 (12)
O3B—C12B—O4B	124.07 (13)	O4D—C13D—C14D	110.86 (13)
O3B—C12B—C4B	124.87 (13)	O4D—C13D—H13A	109.5
O4B—C12B—C4B	111.06 (11)	C14D—C13D—H13A	109.5
O4B—C13B—C14B	110.85 (13)	O4D—C13D—H13B	109.5
O4B—C13B—H13E	109.5	C14D—C13D—H13B	109.5
C14B—C13B—H13E	109.5	H13A—C13D—H13B	108.1
O4B—C13B—H13F	109.5	C13D—C14D—H14A	109.5
C14B—C13B—H13F	109.5	C13D—C14D—H14B	109.5
H13E—C13B—H13F	108.1	H14A—C14D—H14B	109.5
C13B—C14B—H14G	109.5	C13D—C14D—H14C	109.5
C13B—C14B—H14H	109.5	H14A—C14D—H14C	109.5
H14G—C14B—H14H	109.5	H14B—C14D—H14C	109.5
C13B—C14B—H14I	109.5	H5E—O5—H5F	109.3 (16)
H14G—C14B—H14I	109.5	H6E—O6—H6F	104.6 (14)
H14H—C14B—H14I	109.5	H7E—O7—H7F	106.2 (15)
C3C—O2C—H2C	108.5 (13)	H8E—O8—H8F	99.1 (14)
C12C—O4C—C13C	116.09 (11)	H9E—O9—H9F	108.4 (16)
C2C—N1C—C1C	122.91 (12)		

### Hydrogen-bond geometry (Å, º)

**Table:**

*D*—H···*A*	*D*—H	H···*A*	*D*···*A*	*D*—H···*A*
O2*A*—H2*A*···O5	0.81 (2)	1.86 (2)	2.6569 (16)	166 (2)
O2*B*—H2*B*···O1*B*^i^	0.84 (1)	1.89 (2)	2.7111 (14)	165 (2)
O2*C*—H2*C*···O1*C*^ii^	0.85 (1)	1.89 (2)	2.7089 (14)	162 (2)
O2*D*—H2*D*···O8^iii^	0.82 (2)	1.92 (2)	2.7134 (17)	164 (2)
O5—H5*E*···O1*C*^ii^	0.86 (1)	1.94 (2)	2.7857 (15)	168 (2)
O5—H5*F*···O2*D*^iii^	0.86 (1)	1.79 (1)	2.6490 (17)	178 (2)
O6—H6*F*···O2*C*^iv^	0.90 (1)	1.99 (1)	2.8837 (16)	177 (2)
O6—H6*E*···O1*D*^iii^	0.88 (1)	1.96 (2)	2.8218 (15)	167 (2)
O7—H7*F*···O1*A*	0.87 (1)	1.97 (2)	2.8399 (16)	176 (2)
O7—H7*E*···O2*B*^v^	0.89 (1)	2.01 (1)	2.8987 (16)	175 (2)
O8—H8*E*···O7	0.95 (2)	1.87 (2)	2.7637 (18)	156 (2)
O8—H8*F*···O9	0.91 (1)	1.93 (2)	2.8193 (18)	168 (2)
O9—H9*F*···O2*A*	0.84 (2)	1.98 (2)	2.8199 (17)	176 (2)
O9—H9*E*···O6	0.90 (1)	1.93 (2)	2.7724 (18)	155 (2)
C3*B*—H3*B*···O1*A*^vi^	1.00	2.45	3.2581 (17)	138
C3*C*—H3*C*···O1*D*	1.00	2.31	3.1537 (17)	142
C3*D*—H3*D*···O1*D*^iii^	1.00	2.56	3.4252 (18)	145
C5*A*—H5*A*···O7^v^	1.00	2.34	3.2374 (18)	148
C5*B*—H5*B*···O9^vi^	1.00	2.40	3.2568 (19)	143
C5*C*—H5*C*···O8^iii^	1.00	2.49	3.2395 (19)	131
C5*D*—H5*D*···O6	1.00	2.41	3.2010 (18)	135
C7*A*—H7*A*···O3*B*	0.95	2.38	3.2943 (17)	162
C7*B*—H7*B*···O3*A*^vi^	0.95	2.46	3.3253 (18)	151
C7*C*—H7*C*···O3*D*	0.95	2.39	3.2704 (18)	155
C11*A*—H11*A*···O4*B*^iv^	0.95	2.54	3.3027 (17)	138

Symmetry codes: (i) −*x*, −*y*, −*z*+1; (ii) −*x*, −*y*+1, −*z*+1; (iii) −*x*+1, −*y*+1, −*z*+1; (iv) *x*+1, *y*, *z*; (v) −*x*+1, −*y*, −*z*+1; (vi) *x*−1, *y*, *z*.
